# Scaffolds for Dentin–Pulp Complex Regeneration

**DOI:** 10.3390/medicina60010007

**Published:** 2023-12-20

**Authors:** Diana B. Sequeira, Patrícia Diogo, Brenda P. F. A. Gomes, João Peça, João Miguel Marques Santos

**Affiliations:** 1 Institute of Endodontics, Faculty of Medicine, University of Coimbra, 3000-075 Coimbra, Portugalpdn@uc.pt (P.D.); 2 Center for Neuroscience and Cell Biology, University of Coimbra, 3004-504 Coimbra, Portugal; jpeca@cnc.uc.pt; 3 Center for Innovation and Research in Oral Sciences (CIROS), Faculty of Medicine, University of Coimbra, 3000-075 Coimbra, Portugal; 4 Department of Restorative Dentistry, Division of Endodontics, Piracicaba Dental School, State University of Campinas—UNICAMP, Piracicaba 13083-970, Brazil; bpgomes@fop.unicamp.br; 5 Department of Life Sciences, Faculty of Science and Technology, University of Coimbra, 3000-456 Coimbra, Portugal; 6 Coimbra Institute for Clinical and Biomedical Research (iCBR) and Center of Investigation on Environment Genetics and Oncobiology (CIMAGO), Faculty of Medicine, University of Coimbra, 3000-548 Coimbra, Portugal

**Keywords:** dental pulp, guided tissue regeneration, tissue scaffolds, biocompatible materials

## Abstract

*Background and Objectives*: Regenerative dentistry aims to regenerate the pulp–dentin complex and restore those of its functions that have become compromised by pulp injury and/or inflammation. Scaffold-based techniques are a regeneration strategy that replicate a biological environment by utilizing a suitable scaffold, which is considered crucial for the successful regeneration of dental pulp. The aim of the present review is to address the main characteristics of the different scaffolds, as well as their application in dentin–pulp complex regeneration. *Materials and Methods*: A narrative review was conducted by two independent reviewers to answer the research question: What type of scaffolds can be used in dentin–pulp complex regeneration? An electronic search of PubMed, EMBASE and Cochrane library databases was undertaken. Keywords including “pulp-dentin regeneration scaffold” and “pulp-dentin complex regeneration” were used. To locate additional reports, reference mining of the identified papers was undertaken. *Results*: A wide variety of biomaterials is already available for tissue engineering and can be broadly categorized into two groups: (i) natural, and (ii) synthetic, scaffolds. Natural scaffolds often contain bioactive molecules, growth factors, and signaling cues that can positively influence cell behavior. These signaling molecules can promote specific cellular responses, such as cell proliferation and differentiation, crucial for effective tissue regeneration. Synthetic scaffolds offer flexibility in design and can be tailored to meet specific requirements, such as size, shape, and mechanical properties. Moreover, they can be functionalized with bioactive molecules, growth factors, or signaling cues to enhance their biological properties and the manufacturing process can be standardized, ensuring consistent quality for widespread clinical use. *Conclusions*: There is still a lack of evidence to determine the optimal scaffold composition that meets the specific requirements and complexities needed for effectively promoting dental pulp tissue engineering and achieving successful clinical outcomes.

## 1. Introduction

Regenerative medicine is defined as a research field dedicated to the creation of living and functional tissues or organs for medical applications. When applied to dentistry, regeneration may be useful in the repair and mitigation of conditions that arise from damage, absence, or loss of teeth which may result from insults such as trauma, cavities, periodontal disease, fractures, or genetic abnormalities [1,2,3].

Extremely deep carious lesions, dental trauma, periodontal disease and iatrogenic factors can affect the pulp–dentin complex and induce pulp inflammation or necrosis. Traditionally, the most common treatment choice to address this clinical scenario is root canal treatment (RCT), a procedure involving canal debridement, mechanical and chemical preparation and obturation of the canal. The average success rates for RCT in teeth without apical periodontitis varies between 92% to 98%. Nevertheless, this diminishes to 74–86% when apical periodontitis is present [4,5,6,7]. Moreover, following RCT, re-infection of the root canal system and root fracture can occur with significant frequency, leading to necessary non-surgical retreatment, endodontic surgery or tooth loss. The ideal treatment approach involves maintaining the vitality of the pulp in cases of pulpitis and facilitating pulp regeneration for immature permanent teeth, where root development can be paused by pulp necrosis. In these situations, conventional endodontic treatment yields a limited prognosis due to the added challenges posed to the clinician during preparation and obturation. Complete root development with closure of the root apex typically takes place approximately three years after the tooth has erupted into the oral cavity and is in contact with its opposing tooth. The loss of pulp vitality before total root development originates thin and weak dentinal walls that are highly susceptible to fracture [8,9,10]. Thus, efforts should be made to preserve tooth vitality until complete root maturation is achieved.

Tissue engineering (TE) constitutes a part of regenerative medicine that integrates medical engineering, materials science, and applied cell biology to restore or substitute organ function. The most common technique used in tissue engineering consists of seeding cells in a scaffold, these being the 3D structures that the cells can use as an extracellular matrix during a certain period of time to create a favourable environment for their establishment [11]. Ideally, a scaffold should be porous and permeable to facilitate cell migration and nutrient ingress, present appropriate surface for cell attachment, and be both biocompatible and eventually biodegradable into nontoxic products [12]. Because scaffolds provide the template for cells to adhere, proliferate, migrate and spatially organize [13], an ideal scaffold may also be shaped to guide cellular organization. For tooth regeneration, scaffolds require several general characteristics, such as being easy to manipulate, having bioactive and biodegradable properties, adequate porosity, physical and mechanical strength, having low immunogenicity, and being able to support vascularization. In addition to the essential characteristics mentioned earlier, an appropriate scaffold for dentin–pulp regeneration must also have a suitable shape, size, and pore volume to facilitate the penetration and diffusion of growth factors, nutrients, and waste products between cells. This enables effective communication between cells and supports their metabolic needs, aiding in tissue formation and regeneration.

A wide variety of biomaterials are available for tissue engineering, each one presenting particular advantages and disadvantages to be applied in regeneration. Hence, the objective of the current narrative review is to examine the main characteristics of various natural and synthetic scaffolds studied both in vitro and in vivo. The aim is to evaluate their potential clinical application in dentin–pulp complex regeneration.

## 2. Materials and Methods

A narrative review was conducted by two independent reviewers (D.B.S. and J.M.M.S.) to summarily answer the research question: what type of scaffolds can be used in dentin–pulp complex regeneration? An electronic search of PubMed, EMBASE and Cochrane library databases was undertaken until June 2023, without time restriction, including only articles in English language. Suitable keywords, including “pulp-dentin regeneration scaffold” and “pulp-dentin complex regeneration” were used to find papers on tissue engineering approaches. In addition, to locate additional reports, reference mining of the identified papers was undertaken.

Specific scaffolds designed for the regeneration of pulp–dentin complex reported in in vitro and in vivo animal studies were selected, analyzed and synthesized.

## 3. Results and Discussion

A wide variety of biomaterials is available for tissue engineering and can broadly be divided into two categories: natural and synthetic scaffolds, as follows:

### 3.1. Natural and Natural-Derived Polymeric Scaffolds

Natural and natural-derived polymeric scaffolds, as shown in Table 1, namely peptides and polysaccharides, are derived from natural sources and can form hydrogels that possess high water absorbing capacity [14]. They are similar to the native cellular milieu, are highly biocompatible, naturally available and inexpensive. However, due to their biologic nature, these scaffolds present a number of significant limitations, such as batch-to-batch variation and poor mechanical performance [15]. Nonetheless, in many cases, the benefits of using these scaffolds outweigh the drawbacks, rendering them appropriate for regenerating the dentin–pulp complex.

#### 3.1.1. Polysaccharides

Polysaccharides or polycarbohydrates are long chain polymeric carbohydrates composed of monosaccharide units, bounded by glycosidic linkages, which can be hydrolyzed in monosaccharide or oligosaccharides. The most commonly used polysaccharides for dental-pulp regeneration are alginate, cellulose and chitosan.

##### Alginate

Alginate is naturally derived from the cell walls of brown algae (Phaephyceae) or certain bacteria, such as Pseudomonas and Azotobacter. It consists of a linear copolymer of homopolymeric blocks of (1→4)-linked β-D-mannuronate (M) and α-L-guluronate (G) residues, that can be arranged in consecutive single monomers or alternating M- and G-residues, known to form hydrogels [42,43,44]. The gelling properties of this biopolymer are related to interactions with ions such as calcium (via ionic cross-linking), or to low environmental pH value [45]. Physical and chemical properties of alginate are influenced by the M/G ratio as well as by its structural organization [46]. Thus, by increasing calcium levels, the cross-linking density is higher and the alginate gains mechanical strength [47]. Alginates are biocompatible, have low immunogenicity, and exhibit large diversity [48]. However, they also have low mechanical strength and uncontrolled rates of biodegradation [49]. Nonetheless, due to their almost temperature-independent gel state in the presence of multivalent cations, they are suitable for cell immobilization and can be used as hydrogels for various biomedical applications. Alginate hydrogels can be used in dentin–pulp complex regeneration. By adding growth factors such as transforming growth factor beta (TGFβ) or by acid-treating these hydrogels, it is possible to observe differentiation of odontoblast-like cells and regular tubular dentin secretion when applied to cultured human tooth slices [50]. Fujiwara et al. studied the use of alginate as a scaffold in a subcultured rat dental-pulp-derived cells transplantation into nude mice. They found that if beta-glycerophosphate is present, the mRNA of the dentin sialophosphoprotein gene was expressed, as well as alkaline phosphatase, an early marker of odontoblast differentiation. This work showed that subcultured rat dental-pulp-derived cells seeded in an alginate scaffold can differentiate into odontoblast-like cells and can induce calcification [51].

##### Chitosan

Chitosan is a natural linear polysaccharide, composed of randomly distributed β-(1→4)-linked D-glucosamine and N-acetyl-D-glucosamine [52]. Derived from chitin present in fungi, or in the exoskeleton of marine crustaceans and insects [53,54], chitosan is biocompatible, biodegradable and possesses antimicrobial and regenerative properties [16]. An advantage of this scaffold is the possibility for it to bind to growth factors, and also to DNA and glycosaminoglycans [54]. However, controversial results have been re-ported on the use of chitosan as a scaffold for dental pulp stem cells (DPSCs) growth and differentiation. For example, Kim et al. compared the growth and differentiation properties of these cells in three different natural scaffolds: collagen, gelatine and chitosan. Proliferation and differentiation of DPSCs was not appropriately supported in chitosan when compared with gelatine and collagen [55]. However, Feng et al. have reported the successful use of a 3D porous chitosan scaffold on the support, growth and differentiation of DPSCs to nerve cells [56].

Chitosan can be applied as an individual scaffold or in combination with polymers or other biomaterials to produce a large number of matrices for tissue engineering purposes. The addition of chitosan scaffolds into the blood for endodontic regeneration procedures can stimulate the formation of new soft tissue (as proven by histological regeneration) and the formation of mineralized tissue around the pulp canal wall [57]. More recently, chitosan has been added to a cellularized fibrin hydrogel to enhance antibacterial effect, this improved the benefits of this scaffold in a dental pulp regeneration assay [58]. Moreover, incorporation of 2% calcium silicate suspension into a chitosan formulation increased the pore diameter of the scaffold and enhanced calcium release as well as the gene expression of odontogenic markers by human dental pulp cells (hDPCs) [59].

##### Cellulose

Cellulose is the most abundant organic linear polysaccharide. Composed of several hundred to many thousands of glucose units connected by β-1,4-glucosidic linkage [60], it is present on cell walls of green plants, in algae and oomycetes. Cellulose presents excellent biocompatibility, is non-toxic and low cost [61]. Based on these characteristics, the use of cellulose-based hydrogels for biomedical applications has gained attention. However, its poor mechanical properties have limited its application on hard tissue regeneration [62].

#### 3.1.2. Extracellular Matrix Derived

The extracellular matrix (ECM) is a complex network that provides structural and biochemical (i.e., signaling response) support to the surrounding cells [63]. The ECM is mainly composed of extracellular macromolecules that include structural proteins (e.g., collagen and elastin), specialized proteins (e.g., fibrillin, fibronectin and laminin) and glycosaminoglycans (GAGs), hyaluronic acid (HA) and minerals [64]. ECM-based components are produced by resident cells and secreted to surrounding medium via exocytosis [25]. ECM composition can be manipulated for the construction of different types of polymeric and composite scaffolds [16]. ECM scaffolds have gained attention in tissue engineering and regenerative medicine due to their capacity to incorporate and release growth factors. However, batch-to-batch variation and difficulties in processing and sterilizing these compounds present some disadvantages [17].

##### Hyaluronic Acid (HA)

HA is an anionic, non-sulphated GAG, and is present on the extracellular matrix of connective, epithelial and neural tissue. HA is a polymer of disaccharides, composed of D-glucuronic acid and N-acetyl-D-glucosamine [22]. When applied to exposed pulp, HA has been shown to stimulate the production of reparative dentin, aiding in the repair of damaged teeth. HA can be applied in the form of a 3D sponge to create an optimal environment for blood vessel proliferation and stem cell differentiation. This enables the growth of new tissue and the regeneration of damaged tissue, promoting dental pulp revitalization [57]. Scaffolds of HA have important roles for tissue regeneration (cell proliferation and migration), inflammatory response and its degradation products include pro-angiogenic factors. HA hydrogels are biocompatible and display low immunogenic potential [22], but present poor mechanical properties and in vivo degradation kinetics need further improvements to allow complete repopulation of the root canal space by vital tissue [57,65]. In 2010, Inuyama et al. analyzed the behavior of HA sponges seeded with a dental pulp cell line as a scaffold for dental pulp regeneration. In this study, the authors reported a cell-rich reorganizing tissue in the amputated dental pulp region, suggesting that HA sponges are indeed a suitable scaffold for pulp regeneration [66]. Moreover, Silva et al. have investigated HA hydrogels incorporated with cellulose nanocrystals (CNCs) and reinforced with platelet lysate. The incorporation of CNCs remarkably enhanced the stability and mechanical properties of HA hydrogels. It was found that resistance against hydrolytic and enzymatic degradation, the ability to recruit cells, and proangiogenic activity were significantly enhanced by this combination [18].

##### Collagen

Collagen is the most abundant structural protein of the extracellular matrix in mammalian connective tissues and presents the closest viscoelastic properties to real pulp tissue [19]. Collagen is composed of amino acids sequences, typically glycine-X-Y, where X and Y are frequently proline or hydroxyproline, that together form a triple helix. Collagen has multiple applications in medicine, such as cardiac applications, bone grafts or tissue regeneration. Collagen can be extracted from several animal/human sources, such as bone, cartilage, tendon, ligament or skin [23]. Due to its origin, collagen displays low immunogenicity. Collagen is permeable and presents a porous structure, it is also biocompatible and biodegradable [67]. Collagen is involved in regulating cell morphology, adhesion, migration and differentiation [24]. All of these characteristics make this natural polymer a promising biomaterial and scaffold for tissue engineering. However, poor mechanical strength and poor structural stability upon hydration are some disadvantages that can compromise its application [68]. Cross-linking of collagen scaffolds and blending collagen with other materials, such as inorganic materials or natural/synthetic polymers, may provide improvements to achieve better mechanical strength [68,69]. Sumita et al. analyzed the performance of collagen sponge as a 3D scaffold for tooth-tissue engineering. This in vivo study showed that collagen sponge allowed a more reliable tooth generation when compared with a polyglycolic acid fiber mesh scaffold [70]. Additionally, Prescott et al., evaluated the regeneration of dentin pulp-like tissue using DPSCs seeded in a collagen scaffold, with dentin matrix protein 1 (DMP1), when implanted in mice. The study concluded that this triad was sufficient to generate an organized matrix formation of pulp-like tissue [20].

##### Gelatin

Gelatin is composed of peptides and proteins produced by a partial hydrolysis of collagen. Its composition is similar to its parent collagen’s origin. Gelatin is classified as a hydrogel and can be used in food applications, cosmetics, as a carrier, and in cell culture to promote adhesion, among other uses. One particular application is its use in hydrogel synthesis for tissue engineering. Its biocompatibility, low antigenicity, wide availability and low cost are some of the advantages of this natural scaffold [16]; however, gelatin is very sensitive to temperature alterations and degradation over time [21], which may compromise its mechanical properties. Gelatin hydrogels play an important role on cell attachment and provide access to several functional groups for biochemical modification, resulting in a high efficacy scaffold with bio-affinity and improved mechanical properties [71]. For example, Ishimatsu et al. observed dentin regeneration and the formation of dentinal bridges on the surface of regenerated dental pulp, when using controlled release of fibroblast growth factor 2 (FGF2) from gelatin hydrogels [72]. Additionally, Londero et al. performed a histologic analysis of the influence of a gelatin-based scaffold (Gelfoam) in the repair of immature dog teeth subjected to regenerative endodontic treatment, leading to the conclusion that Gelfoam improved tooth repair when combined with blood clot [26].

#### 3.1.3. Proteins and Peptides

Protein and peptide scaffolds are an emerging topic in tissue engineering, due to their versatile structure, composition, and the possibility that they might produce recombinant forms [27]. In addition to biocompatibility and biodegradation, another major advantage of peptides is the possible refinement of their structures via molecular manipulations, allowing the creation of a new modified peptide with specific biological, physical and chemical properties [28].

##### Fibrin

Fibrin is a fibrous non-globular protein, involved in blood coagulation. It is formed by the enzymatic activity of the protease thrombin on protein plasma fibrinogen, causing its polymerization. This scaffold offers advantages in terms of biocompatibility, immunogenicity, cell adhesion, cell proliferation, cell differentiation, biodegradability and cost when compared with other scaffolds [16,73]. Fibrin hydrogels can be obtained via a patient’s own blood, representing an available, reproducible, autologous scaffold, with no immunologic risk. This hydrogel can also be injected and molded to acquire desirable 3D forms [73]. It is degraded by proteases (e.g., plasmin) and metalloproteinases, allowing scaffold redesign and resorption [30]. As with other natural scaffolds, fibrin gels present poor mechanical properties, being susceptible to contraction/compaction [31] and premature degradation [74]. However, these properties can be improved with optimization of the polymerization conditions (pH, calcium/fibrinogen/thrombin concentrations) [75] when fibrin gels are combined with other natural or synthetic polymers, such as HA or calcium phosphate ceramics [30], or when fibrinolysis is controlled with aprotinin, α-aminocaproic acid or tranexamic acid [76,77,78]. Autologous fibrin-rich platelet concentrates and fibrin hydrogels have been applied in dental-pulp regeneration with promising results [79,80,81].

##### Platelet-Rich Plasma (PRP) and Platelet-Rich Fibrin (PRF)

Platelet-rich Plasma (PRP) and platelet-rich fibrin (PRF) are autologous bioactive platelet concentrates prepared ex vivo by centrifugation of a patient’s own blood. These platelet concentrates (PC’s) have been applied in several fields of medicine, such as dentistry, plastic surgery and sports medicine. The use of PC’s is based on improving the healing process and tissue regeneration via the release of biologically active substances (e.g., growth factors) from platelet granules, namely platelet-derived growth factor (PDGF), TGFβ, insulin-like growth factors (IGFs), vascular endothelial growth factor (VEGF), epidermal growth factor (EGF) and epithelial cell growth factor (ECGF) [82,83]. PRP is collected with anticoagulant and is prepared in a simple two-spin centrifugation after which three layers may be collected: (1) the platelet-poor plasma (least dense), representing 45% of the sample; (2) red blood cells (middle layer), representing 40% of the sample; and (3) the PRP (denser layer), representing 15% of the sample. PRP is then mixed with a coagulating agent, such as calcium chloride and/or topical bovine thrombin, to initiate the coagulation process [84,85]. A PRP blood clot generally contains 4% red blood cells, 95% platelets and 1% white blood cells. In PRP, the platelet concentration is five times superior to normal platelet count [86], which increases the amount of growth factors bound to the developing fibrin network or to the extracellular matrix, thus creating a chemotactic gradient for stem cell recruitment, the promotion of tissue healing and regeneration [85]. Despite the advantages of PRP, there is a lack of standardization in PRP preparation protocol, range of storage time of different platelet concentrations and usage of different polymerization strategies [84,85]. Several studies refer to the benefits of using PRP, namely in the treatment of periodontal defects [87], in endodontic regenerative treatment [88,89] and in bone regeneration [29,90].

PRF was developed by Choukroun et al. and is considered a second-generation platelet concentrate. One advantage of PRF over PRP is its “simpler” preparation. After collection, blood is immediately centrifuged without the addition of any anticoagulant [91,92]. In this process there are also three layers in the tube: (1) a top layer consisting of an acellular plasma; (2) a middle layer consisting of PRF; and (3) a coagulation of red blood cells at the bottom. PRF is rich in integrated platelets and leukocyte cytokines. As PRF preparation does not involve the addition of an anticoagulant, the slow fibrin polymerization process favors growth factor entrapment and cell migration [92]. In addition, PRF presents less biochemical alterations and better structural integrity [93]. Limitations of PRF are related with low quantity formation and the loss of structural integrity with time, requiring an immediate use after preparation [94]. Several studies suggest possible applications of PRF on periodontal regeneration [95], regenerative endodontic treatment [96,97] and bone regeneration [34]. Recently, Sequeira et al. embedded human stem cells of the apical papilla (SCAPs) in a platelet-rich plasma (PRP) scaffold and filled empty root segments to assess, in vivo, the new formation of pulp–dentin like tissue. When implanted subcutaneously in an immunodeficient rat, these constructs were able to induce the formation of tubular dentin, with signs of physiologic pre-dentin calcification and pulp tissue with vascular and neuronal components [97].

##### Silk

Silk is a natural protein fiber that can be woven into textiles. It is mainly composed by fibroin and sericin and is produced by certain insect larvae to form cocoons [98]. Silk fibers have been used as suture material for centuries. Gaining attention as a biomaterial, silk has been used as the basis of several structures, such as gels, sponges and films [99]. Silk presents remarkable mechanical properties (high strength, flexibility and toughness), is biocompatible, biodegradable (in vitro and in vivo), and may be processed in water-based solutions. Silk is also cheap, available in high quantities and can be chemically modified to alter side chain surface, to promote cell adhesion or entrapping cellular growth factors [49,98,100]. Mesenchymal stem cells (MSCs) can be seeded on silk biomaterials to achieve specific biological goals. Silk fibroin biomaterials have been applied in wound healing and tissue regeneration, including vascular, neural, bone, skin, and cartilage, among others [101,102]. There are several studies showing the potential use of silk scaffolds in dental regeneration. Xu et al. examined the utility of silk scaffolds on dental tissue engineering, showing that these scaffolds can be useful in forming mineralized osteodentin [103]. Zhang et al. tested human dental pulp progenitor cell behavior on aqueous- and hexafluoroisopropanolol-based silk scaffolds, concluding that alternative materials supported osteodentin and dental pulp formation, in turn suggesting that silk scaffold materials may be appropriate for dental tissue regeneration [104]. More recently, Wei et al. explored the effect of a triple combination of silk–arginine-glycine-aspartic acid (RGD peptide)–stem cell factor on SCAPs. Due to improved stem cell migration, adhesion and proliferation, this study suggests silk-based scaffolds as promising tools for dental pulp regeneration [105].

##### Self-Assembling Peptides (SAP)

Self-assembling peptide (SAP) scaffolds, based on peptide molecules with self-assembling properties, have been investigated since the early 1990s. SAP scaffolds are formed by 15–25 amino acids, that can suffer a spontaneous organization into stable structures and entrap water molecules, forming hydrogels [106]. In 1993, Zhang et al. discovered a 16 residue peptide (EAK16) that spontaneously assembles to form nanofibers, and that, in a salt solution, forms a stable hydrogel [107]. This discovery opened a new research field on different SAP scaffold designs, based on SAPs with different sequences. SAP scaffolds are biocompatible, non-immunogenic, non-toxic and biodegradable, and whose degradation products consist of natural amino acids that can be used by cells [106]. The fact that SAPs undergo a hydration process in physiological conditions, allows for the attachment of cells or bioactive molecules with useful properties for tissue engineering and regeneration [35]. Due to its nanometric structure, it is believed that SAP nanofibers act in a similar way to an extracellular matrix, mimicking a more natural 3D microenvironment. Despite all of these advantages, costs and the complex design parameters represent some limitations of these scaffolds [108].

In 2020, Xia et al. analyzed the influence of an SAP-based scaffold with RGD- and VEGF-mimetic peptide epitopes on the regeneration of the dentin–pulp complex. The authors concluded that the multifunctionalized scaffold promoted cell adhesion and angiogenesis, and stimulated dentin formation and pulp recovery [36].

Galler et al. investigated the use of a customized SAP scaffold seeded with DPSCs and bioactive factors (FGF, TGF-b1, VEGF) in a study that further supported the potential use of these scaffolds in dental pulp regeneration [35].

##### Host-Derived Scaffolds

Host-derived scaffolds are human autologous scaffolds translated in regenerative dentistry as the induction of bleeding and the intracanal blood clot. Both are used to provide a scaffold for pulp–dentin regeneration, as, in immature teeth with open apices, induced bleeding results in SCAP delivery into the canal through the apical foramen with no need for the injection of foreign stem cells to the root. These main principles, when allied with tricalcium silicate-based materials’ simplicity, low costs, short setting time and cervical sealability [109], form an appealing treatment alternative. PRP and PRF are host-derived scaffolds.

Based on the above, the predictability of expected outcomes and the organization of the newly formed tissues are disturbed by the lack of available suitable scaffold that mimics the complexity of the dental pulp extracellular matrix. In 2017, Song et al. proved that decellularized human dental pulp itself supports the proliferation and differentiation of SCAPs [37].

Decellularized ECM scaffolds are derived from natural tissues, wherein cellular components are removed while preserving the essential extracellular matrix structure. This matrix contains various growth factors, signaling molecules, and structural proteins that are critical for cell adhesion, proliferation, and differentiation. Decellularization techniques must effectively remove cellular components while preserving the ECM. Common methods include chemical detergents, enzymatic digestion, and mechanical agitation. Optimizing decellularization methods is critical to maintaining scaffold integrity. An ideal decellularized scaffold is based on matrices compositions that most resemble the composition of the natural ECM structure of the lost tissue, allowing cell infiltration, proliferation, differentiation, and tissue development via the recruitment of autologous MSCs. Dental pulp is just one of several tissue types, including decellularized muscle, submandibular gland, tracheal, cartilaginous, and tooth, that have been decellularized for pulp–dentin regenerative purposes [110].

Shi et al. compared decellularized submandibular gland extracellular matrix (DSMG) with decellularized human dental pulp in vitro and in vivo, confirming that DSMG could support adhesion and proliferation of dental pulp stem cells. Implantation of cell-seeded DSMG in an in vivo model allowed the formation of a vascularized dental pulp-like tissue, with similar results for both of the decellularized tissues studied. The submandibular gland is a vital maxillofacial organ with an abundant ECM and basic matrix proteins similar to dental pulp. This novel ECM can overcome the limitations associated with the small amount of pulp tissue available for harvesting in humans or animals, providing an accessible and effective alternative [111].

Numerous animal studies have demonstrated the regenerative potential of ECM scaffolds in dental pulp repair. These studies often report improved tissue formation, angiogenesis, and the development of a new vascular network within the tooth [110]. While significant progress has been made in preclinical studies, further research is needed to refine scaffold properties, assess long-term safety, and advance toward clinical applications. Treated dentin matrix (TDM) is an autologous scaffold with diverse nonmineralized dentin matrix components such as glycosaminoglycan, chondroitin sulfate, type I collagen, bone morphogenetic protein, dentin sialoprotein, amid others. In 2021, Wen et al. recommended TDM as a potential bioactive pulp-capping material for vital pulp therapy, as TDM effectively recruits DPCs, induces the odontogenic process, and stimulates the formation of reactive dentin leading to a complete dentin bridge regeneration with no tuned defects [112]. Furthermore, when TDM is combined with small extracellular vesicles (sEV), the TDM-sEV complex exhibits intrinsic biological activities and, in comparison to MTA, has more suitable odontogenic inductivity (in a mini-pig model).

### 3.2. Synthetic-Engineered Polymeric and Ceramic Scaffolds

#### 3.2.1. Synthetic Polymeric Scaffolds

Synthetic polymers constitute the largest biodegradable polymer group. Produced under controlled conditions, synthetic polymeric scaffolds display predictable and reproducible properties (e.g., mechanical characteristics, viscosity, porosity, biodegradation). These have the advantage of being tunable (i.e., in terms of their physical and chemical properties) and can be designed to release growth factors or other bioactive molecules. They can be produced in large quantities, may be more economical than natural scaffolds and present a longer shelf life [38]. The major disadvantage of these scaffold groups is the limited bioactivity due to their hydrophobic structure [113]. Among the most popular synthetic polymers used in tissue engineering are polylactic acid (PLA), polyglycolic acid (PGA) and polylactide-co-glycolide (PLGA) [114]. PLA and PGA are, respectively, polymers produced by lactic acid and glycolic acid condensation. Their main benefit for medical applications resides in the fact that their degradation products are natural metabolites, normally excreted in urine [115]. However, concerns about PLA/PGA biocompatibility have been raised due the accumulation of the degradation products [116]. PLGA is a copolymer composed of two different monomers, the cyclic dimers of glycolic acid and lactic acid. The ratio between glycolic and lactic acid leads to the formation of different forms of PLGA, with different degradation times [16]. These synthetic polymeric scaffolds have been used in dentistry for dental-pulp tissue regeneration and shown to be amenable to the seeding of DSCs. Early studies by Mooney et al. have described the formation of new pulp-like tissues when dental pulp stem cells (DPSCs) were seeded onto fabricated PGA fibers [117]. Later, Kuang et al. produced biocompatible and biodegradable PLA-based scaffolds and assessed their regulatory role in dentin-pulp complex regeneration. The PLA-based scaffolds considerably promoted the proliferation and odontogenic differentiation of DPSCs ameliorating the expression of ALP, osteocalcin, bone sialoprotein, collagen 1, and dentin sialophosphoprotein genes in an in vitro experiment. Moreover, histological analysis demonstrated superior dentin-like tissue formation in vivo [118].

PLGA scaffolds were also shown to lead to increased proliferation and adhesion of DPSCs under simulated microgravity—a procedure which also enhances MSC growth [119]. Recently, using SHED, PLA scaffolds demonstrated both a good biocompatibility and the ability to induce mineralization [120]. The copolymers of PGAs and PLAs have been sown with dental pulp progenitor cells and have been shown in rabbit and mouse xenograft models to produce pulp-like tissue [109].

#### 3.2.2. Bioactive Ceramic Scaffolds

Bioactive ceramics include calcium phosphate ceramics, bioactive glasses and glass ceramics. These are biocompatible inorganic non-metallic materials that have been widely used in tissue engineering and regeneration, namely bone implants and dentistry [121]. These compounds are known for their good resistance, although the major limitations include brittleness, poor mechanical properties (fracture strength and reliability), low resilience and high density [38].

#### 3.2.3. Calcium Phosphates (CaP)

Synthetic calcium phosphate (CaP) materials like hydroxyapatite (HAP), tricalcium phosphate (TCP) and biphasic calcium phosphate (HAP/TCP) are frequently used as bone grafting materials due to their similarity to the bone mineral phase [32]. These materials are biocompatible, have low immunogenicity, present good properties of resorption and are osteoconductive [33,122]. Properties of CaP ceramics, such as porosity, degradation and ion release, affect the bioactivity of these scaffolds, namely adhesion, proliferation and differentiation of cells [122,123]. High degradation, together with calcium and phosphorus ion release regulate osteoblast and osteoclast activity, promoting the formation of bone minerals in the surface of CaP scaffolds [124,125,126,127]. The bioactivity properties vary according to the physical and chemical characteristics of the CaP material [124]. To improve their advantages and complement their limitations, CaP have been combined or mixed with other materials.

#### 3.2.4. Hydroxyapatite (HAP)

HAP is a naturally occurring mineral form of calcium apatite, with a Ca/P ratio of 1.67, constituting 70% *w*/*w* of human bones [128,129]. Synthetic HAP is produced as a dense material that mimics the mineral composition of bone; however, porous HAP has been used in clinical applications, namely for bone regeneration. HAP scaffold characteristics, such as porosity and pore size, influence its performance [130]. HAP is the most stable calcium phosphate, presenting low solubility in physiological medium, and its surface can function as a nucleating site for bone mineral formation [123,131]. Over the years, studies in vitro and in vivo have demonstrated good biocompatibility, bioactivity, and osteoconductivity for HAP [132,133,134]. However, the brittleness of this scaffold has limited its application when high loads are present.

Nevertheless, HAP is widely used in the coating of other materials (e.g., metal implants) [135,136] and electrospun composite scaffolds made of polycaprolactone/gelatin and nanohydroxyapatite enhanced the proliferation and odontogenic differentiation of DPSCs [137].

#### 3.2.5. Tricalcium Phosphate (TCP)

TCP is a calcium phosphate and a bone substitute material with a Ca/P ratio of 1.5. It is characterized by a high biocompatibility, good resorption properties and osteoconductivity [138]. TCP is available in two forms: α-TCP (formed at ≥1125 °C) and β-TCP (formed at 900–1100 °C). β-TCP presents higher structural stability and higher degradation rate when compared with α-TCP [124]. For these reasons, β-TCP is more widely used in bone regeneration applications [139]. In comparison to HAP, β-TCP is less stable, however it presents higher solubility and higher degradation/resorption rates during bone regeneration [140,141]. All of these characteristics have turned attention to β-TCP as a scaffold for bone regeneration [142,143].

#### 3.2.6. Biphasic Calcium Phosphate (HPA/TCP or BCP)

HAP/TCP or biphasic calcium phosphates were developed to unite, at a submicron level, characteristics from both HAP and TCP—more stability from HAP and better resorption from TCP [144]. Because HAP:TCP ratios influence the ceramic performance, Arinzeh et al. compared the influence of different ratios of biphasic calcium phosphate ceramics combined with MSCs on bone formation. The authors concluded that a HA/TCP formula with higher quantities of TCP induced osteogenic differentiation and bone formation at a faster rate [145]. In 2010, Tonomura et al. tested the differential effect of scaffold shape on dentin regeneration. Their results show that, when porous HAP/β-TCP was used, dentin-like tissue with minimum cell inclusions was observed and aligned odontoblast-like cells appeared in relation to the hard tissue. In HAP/β-TCP powder and PGA groups, bone-like tissue with cell inclusions was observed with no cell alignment. Interestingly, the authors of this study were also able to conclude that the shape of the scaffold influenced the type of tissue regenerated [146].

#### 3.2.7. Bioactive Glasses and Glass Ceramics

Glass is a non-crystalline amorphous solid that has been used by humans for thousands of years, first in tools and arrow heads, and more recently in objects, optical instruments, and fibers [147]. The first bioactive glass, named Bioglass^®^ 45S5, was developed by Hench in 1969 [148]. Bioactive glasses are a group of glasses constituted by different combinations of oxides (e.g., silicon dioxide, sodium oxide, calcium oxide, phosphorus pentoxide, magnesium oxide and ferric oxide), that present a reactive surface which is free to interact with the surrounding environment and establish a strong interface interaction [16,147]. The biocompatibility and bioactivity of these materials has led them to be used in medical applications, namely, orthopedics [41]. These biomaterials react with body fluids, forming a bone-like apatite layer on their surface, and release ions that may promote bone regeneration [41,149]. The apatite layer stimulates cell adhesion and osteogenic cells proliferation, meaning that this layer is substituted by bone tissue over time [150]. Despite excellent biocompatibility and bioactivity, these materials present low mechanical strength and fracture toughness, restricting their application. To overcome these limitations, novel manufacturing techniques have been developed, and bioactive glasses have been combined with polymeric phases, producing composite materials [41]. In dentistry, bioactive glasses and glass ceramics are used as veneers and crowns/bridges, as well as scaffolds for tissue regeneration [151]. El-Gendy et al. evaluated the osteogenic differentiation of human DPSCs on 45S5 Bioglass^®^-based scaffolds in vitro and in vivo. They observed that this scaffold promoted osteogenic differentiation of DPSCs, which led the authors to propose this as a promising candidate for bone tissue regeneration/repair [152].

#### 3.2.8. Composite Scaffolds

Composite scaffolds constituted by biopolymers and bioceramics have become a promising option for dentin–pulp regeneration due to the balance between the advantages and limitations of each individual component. The combination of natural and synthetic materials results in composite materials with improved properties, such as increased biocompatibility, better mechanical strength, and enhanced biological functionality [153]. For instance, Chiu et al. have investigated the MTA properties when combined with a polycaprolactone hybrid 3D scaffold seeded with DPSCs for tissue regeneration. They found that this composite scaffold improved DPSCs adhesion, proliferation and differentiation, suggesting a potential use in tissue engineering [154].

In another study, Vera-Sánchez et al. demonstrated the benefits of silk-fibroin and graphene oxide composites as a scaffold for periodontal ligament stem cells (PDLSCs). They found that the composite scaffold supported good cell adhesion and proliferation and promoted of osteo-/cementoblast differentiation [39]. Moreover, Xie et al. showed that graphene can improve the physicochemical and mechanical properties of composite scaffolds when combined with other materials. This material has potential to be used as a scaffold in combination with other biomolecules or biomaterials [40]. Recently, Zafeiris et al. synthesized a hybrid HAP hydrogel, consisting of HAP nanocrystals, produced in the presence of chitosan and l-arginin. They used genipin, a natural crosslinking agent, to improve the mechanical properties of the hydrogel. This hybrid scaffold not only showed good biocompatibility but also presented suitable characteristics for 3D bioprinting [155]. The present review aimed to discuss strategies centered around scaffolds, summarizing and evaluating the existing state of research on scaffolds applied to the regeneration of the dentin–pulp complex. This emerging research area is currently situated at the preclinical stage, with several approaches under investigation and numerous future perspectives in this domain. In the near future, additional clinical studies are necessary to assess the outcome associated with the use of different types of scaffolds for dentin–pulp complex regeneration.

## 4. Conclusions

The examination of articles published in this field demonstrates an increasing number of studies focused on improving the natural and synthetic scaffolds in order to achieve the conditions required for the regeneration of a functional pulp–dentin complex. Based on the results of this review, the evidence currently available is based on preclinical, in vitro and in vivo studies, and hence lacks the ability to pinpoint the optimal scaffold structure to effectively meet the particular demands and intricacies crucial for advancing pulp–dentin tissue engineering. Nonetheless, the development of a regenerative strategy using advanced scaffolds, loaded or not with stem cells and/or growth factors, to stimulate pulp and dentin regeneration after attaining an adequate niche, is warranted in order to establish novel therapeutics to treat teeth with necrotic pulp.

## Figures and Tables

**Table 1:** Scaffolds used in tissue engineering.

Material	Advantages	Limitations	References
Natural and Natural-Derived Polymeric Scaffolds			
**Polysaccharides**	Derived from renewable sources	Batch-to-batch variation	[14,15]
	Biocompatibility	Poor mechanical properties	
	Low cost		
Alginate	Biocompatibility	Low mechanical strength	[16,17]
	Low immunogenicity	Uncontrolled biodegradation rate	
	Degradation by enzymolysis		
	Large diversity		
Chitosan	Biocompatibility	Allergies	[18,19]
	Biodegradable		
	Antimicrobial potential		
	Regenerative properties		
	Ability to bind GF, glycosaminoglycans and DNA		
	Different forms		
Cellulose	Biocompatibility	Biodegradation in humans (limited or absent)	[20,21]
	Non-toxic	Poor mechanical properties	
	High tensile strength		
	Pliable		
**Extracelullar Matrix Derived**	Dynamic environment	Batch-to-batch variation	[16]
	Composition can be adjusted	Processing and sterilizing difficulties	
	Capacity to incorporate and release growth factors		
Hyaluronic acid	Biocompatibility	High degradation rate	[17,22]
	Low immunogenicity	Poor mechanical properties	
Collagen	Biocompatibility	Poor mechanical properties upon hydration	[23,24]
	Low immunogenicity	Difficult to customize	
	Osteoblastic differentiation stimulant		
	Easy placement of cells and GF		
	Natural replacement after degradation		
Gelatin	Biocompatibility	Sensitive to temperature alterations	[20,25]
	Low antigenicity	Degradation over time	
	Wide availability		
	Low cost		
	Access to several functional groups for biochemical modification		
**Proteins and Peptides**	Dynamic environment	Processing and sterilizing difficulties	[26,27]
	Biocompatibility		
	Biodegradation		
	Provide chemical signals to guide cell behavior		
	Possible refinement of their structures with molecular manipulation		
Fibrin	Injectable and molded to acquire desirable 3D forms Reproducible	Poor mechanical properties—low mechanical stiffness	[19,28,29,30,31]
	Low cost	Rapid degradation	
	Autologous source—no immunologic risk		
Silk	Biocompatibility	Irritant sericin coating	[17,32,33]
	Biodegradable		
	Non immunogenic		
	Low cost		
	Available		
	Remarkable mechanical properties		
	Different forms		
Self-assembling peptides	Biocompatibility	High cost	[34,35]
	Biodegradable	Complex design parameters	
	Non immunogenic		
	Easy to use (injectable)		
	Nanometric		
	More natural 3D microenvironment		
Host-derived scaffolds	Autologous source—no immunologic risk	Specific equipment and reagents are mandatory.	[36]
Platelet-Rich Plasma (PRP)	Favorable for tissue growth		
Platelet-Rich Fibrin (PRF)	Controlled growth factor release		
Decellularized extracelullar matrix (ECM)	Adaptable into specific shapes		
Treated dentin matrix (TDM)	Low costs		
**Synthetic-Engineered Polymeric and Ceramic Scaffolds**			
**Synthetic Polymers**	Biocompatibility	Lack physiological and chemical information	[37,38]
Polylactic acid (PLA)	Mild inflammation		
Polyglycolic acid (PGA)	Low cost		
Polylactide-co-glycolide (PLGA)	Reproducible		
	Tailorable mechanical properties		
	Biodegradable—degradation products are natural metabolites		
**Bioactive Ceramics**		Brittleness	[37,39,40]
**Calcium Phosphates**	Biocompatibility	High density	
**Hydroxyapatite (HA)**	Low immunogenicity	Low resilience	
**Tricalcium Phosphate (TCP)**	Osteoconductivity	Poor mechanical properties	
	Good resistance		
**Bioactive Glasses**	Surface apatite layer formation	Poor mechanical properties	[41]
	Stimulates osteoblastic activity	Brittleness	
		Density	
		Low degradation rate	
